# SLDMS: A Tool for Calculating the Overlapping Regions of Sequences

**DOI:** 10.3389/fpls.2021.813036

**Published:** 2022-01-03

**Authors:** Yu Chen, DongLiang You, TianJiao Zhang, GuoHua Wang

**Affiliations:** ^1^College of Information and Computer Engineering, Northeast Forestry University, Harbin, China; ^2^State Key Laboratory of Tree Genetics and Breeding, Northeast Forestry University, Harbin, China

**Keywords:** algorithm, sequence analysis, genome assembly, contig assembly, overlapping regions, application

## Abstract

In the field of genome assembly, contig assembly is one of the most important parts. Contig assembly requires the processing of overlapping regions of a large number of DNA sequences and this calculation usually takes a lot of time. The time consumption of contig assembly algorithms is an important indicator to evaluate the degree of algorithm superiority. Existing methods for processing overlapping regions of sequences consume too much in terms of running time. Therefore, we propose a method SLDMS for processing sequence overlapping regions based on suffix array and monotonic stack, which can effectively improve the efficiency of sequence overlapping regions processing. The running time of the SLDMS is much less than that of Canu and Flye in dealing with the sequence overlap interval and in some data with most sequencing errors occur at both the ends of the sequencing data, the running time of the SLDMS is only about one-tenth of the other two methods.

## Introduction

Due to the limitations of existing gene sequencing technology, we cannot directly obtain the entire gene sequence, but can only use existing sequencing methods to sequence the genes of the species to be tested to generate sequence fragments and then further genome assembly to restore the original genes. The genome assembly problem is also one of the most important and difficult problems in bioinformatics today.

The two algorithms commonly used in genome assembly are the overlap-layout-consensus (OLC) (Li, 2012) algorithm and the de-bruijn-graph (DBG) (Li, 2012) algorithm, which use different methods to convert the assembly problem into a graph-theoretic related problem. By creating an edge-weighted graph of the sequencing data, the resulting edge-weighted graph is processed to find relevant pathway information in the graph for use in downstream genome assembly work. All the algorithms derive the optimal path from the edge-weighted graph to obtain the initial contig (Huang, 1992).

Most applications for genome assembly are based on one of the algorithms such as the Canu (Koren et al., 2017), which chooses to use the MHAP (Koren et al., 2017) algorithm to detect the overlap in noisy sequences to obtain the overlapping regions between sequences. Additionally, the Flye (Lin et al., 2016) software uses the ABruijn (Lin et al., 2016) algorithm to combine the OLC and DBG algorithms, generates its own unique A-bruijn-graph (ABG) graph, and obtains the overlapping regions of the nodes in the graph and some other assembly software can also complete the same work. These software are usually more time-consuming in the sequence alignment process. For example, Flye takes longer to find overlapping regions of sequences and Canu is slower to correct sequencing data, etc.

This article presents a new software for overlapping regions calculation called the SLDMS, a tool that uses gene sequencing data as input and supports the fastq and fasta formats. It can calculate an output overlapping regions information between sequencing data and write it to a file, so that other applications can use it. Compared to other genome assembly software that calculates overlapping regions, our monotonic stack and suffix array-based design approach is more efficient and provides richer pathway information for downstream genome assembly software to use as a reference. At the same time, the SLDMS can be easily integrated into the genomic analysis process.

## Methods

**FIGURE 1:**
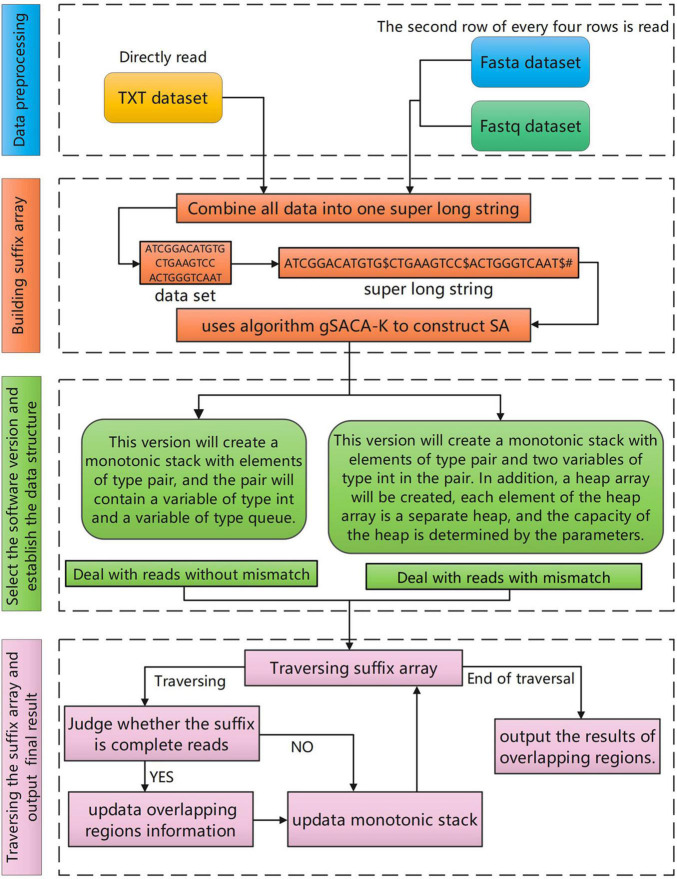
The overall workflow of the SLDMS.

The overall workflow of the SLDMS (Figure 1) includes four steps: (i) data preprocessing; (ii) building a suffix array; (iii) selecting the software version and establishing the relevant data structure; and (iv) traversing the suffix array and output the results of overlapping regions.

### Data Structure

The SLDMS needs three arrays when obtaining overlapping regions information, These three arrays are the suffix array (SA) array (Manber and Myers, 1993) longest common prefix (LCP) array (Fischer, 2010), and Document array (DA) array (Muthukrishnan, 2002). First, we briefly introduce these three arrays. The SA array is the suffix array and SA(i) represents the starting position of the suffix whose string rank is i in the original string. The LCP array is the longest common prefix array and LCP(i) represents the longest common prefix of the suffixes represented by SA(i) and SA(i-1). The DA array is a document array. DA(i) represents the number of strings in the input data to which the suffix ranked i belongs to. This array can be obtained in the process of obtaining SA and LCP.

**FIGURE 2:**
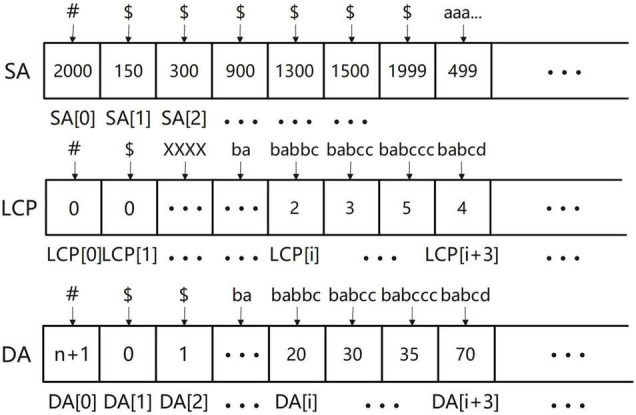
The elements stored in three arrays.

The meaning of the elements stored in the three arrays is given in Figure 2. The SA array: The string above the array is the suffix represented by each item in the array and the value stored in the array is the starting position of the suffix it represents in the original string. LCP array: The string above the array is the suffix represented by each item in the array and the value stored in the array is the length of the longest common prefix between the suffix it represents and the suffix ranked one place ahead of it; to calculate this length, we ignore the ending symbols of the suffix. The DA array: The string above the array is the suffix represented by each item in the array and the value stored in the array is the source of the suffix. For example, DA(i) = 20, “babbc” belongs to the 20th input sequence.

### Algorithm Principle

**FIGURE 3:**
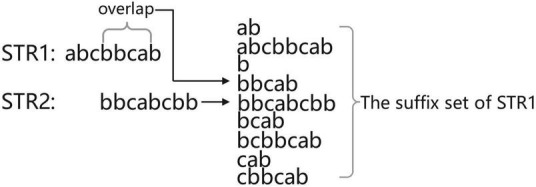
The position of str2 in the suffix set of str1.

We define read as a piece of data in the sequencing data and its representation in the computer is a string. Before finding the overlapping regions information of the sequencing data, first consider the case of finding the overlapping regions information for two reads. Suppose the two reads are str1 and str2; if the tail of str1 and the head of str2 overlap and the overlap starts at position i, then the suffix suf [suf = str1(i:)] of str1 must be the same as some prefix of str2. In other words, the overlap part is the prefix of str2 (otherwise, str2 is the substring of str1 and the splicing is equivalent to discarding str2, so there is no need to splice str1 with str2 in this case), so the longest overlap part of str1 and str2 must be a suffix belonging to str1 that is ranked before str2 in the dictionary order. The overlapping regions information can be obtained by sorting all the suffixes of str1 with str2 and processing the suffixes ranked before str2 (Figure 3). As shown in Figure 3, the set of suffixes in Figure 3 does not show suffixes belonging to str2 because read cannot be connected to itself, so it will make a judgment on the belonging of suffixes and ignore these suffixes belonging to itself when calculating the candidate answers of str2.

Extend the case of two reads overlapping to a set of reads. All the suffixes of the reads are sorted, the best overlapping regions information of each read must exist in some suffix ranked before it, and the read to which this suffix belongs is the maximum possible adjacent node of the current read after building the graph.

Since reading itself is also a suffix of reads, when traversing the set of suffixes, if we encounter a certain read itself, we are able to guarantee that the suffix with its optimal overlapping regions information must have been traversed. Then, the problem is transformed into that the longest common prefix, which is calculated for all the suffixes ranked before it and the current string and when the length of the common prefix is equal to the length of this suffix, we consider this suffix as a candidate answer and select the best or top-K optimal as the final answer among all the candidate answers.

In the design of the algorithm, we choose two advanced data structures, suffix array, and monotone stack, because the information stored in the suffix array is the suffix of the string sorted in dictionary order, which corresponds to the suffix set in Figure 3. The reason for choosing the monotonic stack is that the monotonic stack can work with the LCP array to filter the set of suffixes and remove those suffixes that are not likely to be the answer and improve the computation speed in this way. When we get a certain candidate answer with length *x*, the rest of the candidates with length greater than *x* must not match exactly with the subsequent reads; this is because their common prefixes have a maximum length of *x*, so these candidates should be removed. The monotonic stack exists to remove this part of information.

## Implementation

### Constructing the SA Array, LCP Array, and DA Array

Since the method of constructing the SA and LCP arrays in a single string is quite mature, the SLDMS stitches all the reads in the data into a single string, splitting the sequence with American Standard Code for Information Interchange (ASCII) code 1, and ending the stitching with ASCII code 0. This allows the read set to be treated as a string and we call this stitched together extra-long string the “original string.” For this part, we use gsufsort (Louza et al., 2020) software based on the gSACA-K (Louza et al., 2017) algorithm to obtain the three arrays.

### Maintaining the Monotonic Stack

Assuming that the number of reads is *n*, after obtaining the information of the SA array and LCP array, the first n + 1 items of the SA array are traversed. These n + 1 items are the positions of the interval $ between strings and the string terminator # in the original string. Therefore, we can obtain the start and end positions of each read in the original string and record them in the Fi array and Se array. For example, the start position of the xth read is Fi(x) = SA(X − 1) + 1 and the end position is Se(x) = SA(x). According to the start position and end position of each read, its length was also calculated as LEN(x) = [SE(x) − Fi(x) + 1].

After obtaining the above information, the matching process of suffixes and reads can be optimized by maintaining a monotonic stack. For different input data, different strategies are adopted and the SLDMS was designed in two versions. The first version considers the data to be completely correct and can directly perform the overlapping regions calculation. The accuracy of the result depends on the input dataset and if the dataset is completely correct, the result is also completely correct. Therefore, the input data required to use this version should be either corrected high-accuracy data or raw high-accuracy data such as the PacBio-HiFi (Hon et al., 2020) dataset and the Sanger dataset. The second version allows some differences between reads when performing overlapping regions calculations. Overlapping regions information can be obtained for data with some errors, the accuracy of the information fluctuates depending on the characteristics of the errors in the data, and the accuracy of the results of the runs varies from one dataset to another. After experiments, it is found that the accuracy of the run results is greatly improved when the error part in the reads is gathered at both the ends. Therefore, for the second version, if the errors in the dataset are completely random, it is recommended to correct the complete data first before using the first version or correct the data center part first before using the second version of the software.

For the part of data error correction, we suggest that the third-generation sequencing data PacBio can be used for self-error correction (Hon et al., 2020) or the second-generation sequencing Illumina data can be used for error correction of the third-generation sequencing data PacBio (Mahmoud et al., 2017) such as PBCR (Koren et al., 2012) in the famous Celera Assembler (Schatz, 2006; Denisov et al., 2008) software and LoRDEC (Leena and Eric, 2014) error correction tool. For the datasets with some regularity of data errors (the errors of the sequencing appear on both the sides of the reads), the second version of this software can be chosen directly.

### Deal With Reads Without Mismatch

In this version, since the data can maintain a high accuracy rate, it is enough to directly obtain the overlapping regions information of the reads and the problem of error correction of the reads is not involved. The algorithm idea is as follows.

Build a monotonic stack. The stack is implemented by array simulation to facilitate the acquisition of data in the stack. The element type stored in the stack is a structure similar to a pair designed by us. Its first element is of the int type, which is used to represent the length of the suffix stored in the current element. Its second element is a rolling array, which is used to store the DA information of the suffix that meets the first condition. The length of the array can be set artificially; for example, the length of the array is set to *n*, i.e., to store the top n best answers for each read. In this way, we can obtain more overlapping information between reads. The struct design of the monotone stack and stack elements is found in Supplementary Figure 1, where vector is the structure of the stack, pair is the element stored in stack, and queue is the main part of storing information in the pair, which is realized by a rolling array (Supplementary Figure 2).

**FIGURE 4:**
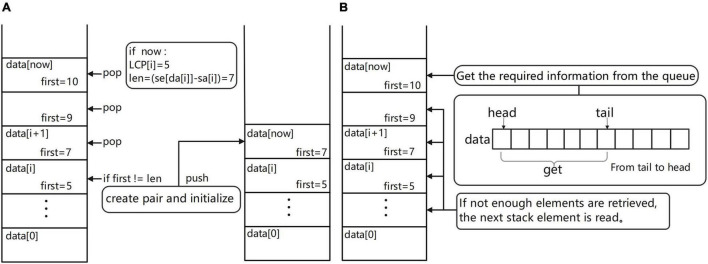
**(A)** Schematic diagram of the monotonic stack maintenance process. **(B)** Diagram of the process of obtaining overlapping regions information.

Maintain a monotonous stack (Figure 4A). Let one suffix ranked Y be str and for all the suffixes ranked before Y, assuming their rank is X, their longest common prefix with str, i.e., the length of LCP, must be equal to min[LCP(X + 1:Y)]. According to this property, when traversing the SA and LCP arrays, each time a new LCP(i) is traversed and the elements of the stack whose first item is larger than LCP(i) can be taken off the stack because for these elements and the following suffix the LCP cannot be greater than LCP(i), so these suffixes become useless information and can be cleaned up. Start to operate the elements in the monotonic stack from the top of the stack; if the first element at the top of the stack is larger than LCP(x), just get this top element out of the stack directly and loop this operation until it is impossible to get out of the stack. After clearing, check the pair at the top of the stack, whether its first element is equal to the length of the string corresponding to the current SA(i) [len = se(DA(i) – SA(i))], if it is equal, put DA(i) into the second scrolling array of the pair and update the array. If it is not equal, create a new pair element whose first is equal to len and whose initial values in the second element are set as follows: head = 0, tail = 1, have = 1, size = k, and data(0) = DA(i). After the element is created, this element is put on the stack. Since all the elements in the stack whose first is greater than len have been removed before entering the stack, each element entering the stack must be the largest element in the stack, so the monotonicity of the stack can be guaranteed, which is exactly the reason for using a monotonic stack.

Get overlapping regions information (Figure 4B). In the process of maintaining the monotonic stack, if the current suffix is an ordinary suffix, just follow the normal process of maintaining the monotonic stack and if the current suffix is a complete read [the value of SA(i) is equal to fi(DA(i))], then we should add the process of information acquisition to the normal maintenance process; at this time, you can maintain the monotonic stack information to obtain the required overlapping regions information. The way to obtain the required overlapping regions information is very simple; first of all, we must first check the top of the stack elements to ensure that the top of the stack elements are not expired (if the elements are expired, they can be taken out of the stack). Then, read the data from the top of the stack and read the second item of each element; these are the reads that are most likely to overlap with the current read and output the overlapping regions information to the result file for use in building the edge weight graph. By default, the first ten possible results are provided, the longest overlapping regions read is usually selected, and the specific read chosen as the path in the edge weight graph can be freely chosen according to the subsequent software requirements.

### Deal With Reads With Mismatch

The first-generation sequencing data are high-accuracy data, but they are no longer in mainstream use because of their expensive sequencing price. The second-generation sequencing data are short-read data with high accuracy and are more suitable for use with DBG assembly software based on K-mer counting (Wang et al., 2020) such as the SOAPdenovo (Xie et al., 2014) software. The third-generation sequencing data are long-read data with a high error rate and there would be a high error rate if the sequencing data was matched exactly, so this version allows for slight differences in sequence during the matching process. This version is an alternative solution to cope with the situation in which the data cannot be completely corrected because of the long correction time or high correction cost of the dataset.

In this version, the error part of the input data should appear at both the ends of the data as far as possible or the data center part has been corrected to ensure that there will be no error in the middle part of each read. The closer the error location is to both the ends, the better the overlapping regions information will be. In this version, a parameter K will be entered, which determines the maximum allowable cutting size of the sequence head and tail when the algorithm is matching. If K is set speculatively without knowing much about the dataset, there may be some error in the result obtained from a single run due to the parameters. But, the speed of fault-tolerant matching is very fast, we can get the final result by inputting different fault-tolerant parameters and running this version many times. We can also get the result at one time by accurately setting K on the basis of knowing the error distribution of dataset. The optimal value of K is set to ensure that the error data at both the ends can be excised on the basis of as small as possible. The algorithm idea is as follows.

Build a monotone stack. The monotone stack in this version chooses to use another structure similar to a pair. Its first element stores X characters in all the previous reads within the fault tolerance range that match the current suffix. This first element represents X. The second element is no longer a rolling array, but represents the sequence number of the suffix with length X in the suffix array within the fault tolerance range. The final result is obtained by maintaining and optimizing all the second elements in the monotone stack whose first elements are greater than LCP(i). The concept diagram of the monotone stack is shown in Supplementary Figure 3.

**FIGURE 5:**
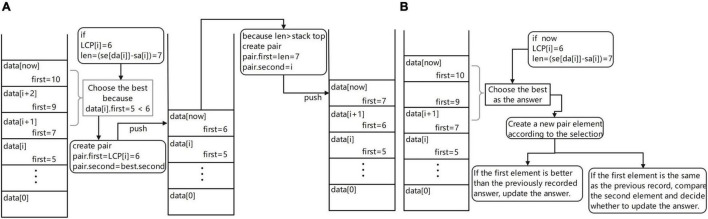
**(A)** Maintenance process diagram of monotone stack and **(B)** Overlap regions information acquisition flow diagram.

Maintain the monotonous stack (Figure 5A). Different from the previous version, if the first element at the top of the stack is greater than LCP(x), it does not directly take the element at the top of the stack out of the stack, but takes out all the stack elements whose first element is greater than or equal to LCP(x) and selects the best element after comparison to access back to the top of the stack. The element that meets the condition [len(STR) – LCP(x)] is the best and its first item is assigned to LCP(x) and its second item to the second of the optimal element. This process is equivalent to allowing an excision operation at the end of the read, where the wrong part is excised and matched again and the length of the allowed excision is set by the user of the software. After updating the stack, use the current suffix information to create a new pair element. If the length is larger than the top of the stack, put it on the stack. If the length is the same, replace the top of the stack. Of course, to prevent self-loops in the graph constructed from the last obtained overlapping regions information, the stack update is performed by ignoring the suffix of the read itself.

Get overlapping regions information (Figure 5B). When traversing the suffix array under the previous version, the result is obtained only when the current suffix is a complete read. In this version, the result is obtained when the first character of the current suffix is the first K characters of the original string, but of course K is determinable and this operation is equivalent to allowing the head of the sequencing data to be cut and the allowed cut length is K. Each cut method is tried, so that each read is compared several times and stores the maximum possible result. Therefore, two auxiliary arrays are needed for multiple result comparisons, the ANS array and the LEN array, where the ANS array stores the ordinal number of its result in the SA array and the LEN array stores the matched lengths. In the process of maintaining the monotonic stack, if the current suffix is the first K suffixes of the string to which it belongs, it is compared with the top element of the stack and the better result is stored. The final ANS array is obtained after several maintenance sessions. After traversing the SA array under this version, the resulting DA[ANS(i)] is the optimal overlapping regions information to be obtained.

Maintain overlapping regions information. To facilitate subsequent assembly software, the SLDMS provide more overlapping regions information for subsequent software. In this version, a top-k overlap suffix set is also provided for each sequence to facilitate subsequent work on genome assembly and parameters are required to set k before running the software. The data structure used to maintain this set of suffixes is the min heap and the top of the heap stores the Kth good overlapping regions (Supplementary Figure 4). The reason for choosing this data structure is that the heap can efficiently maintain the largest or smallest value in the heap, so a min-heap of capacity K is created and the top of the heap is the worst quality of the candidate answer and when a new candidate answer is encountered, it only needs to be compared with the top of the heap, which facilitates the update of the answer.

**FIGURE 6:**
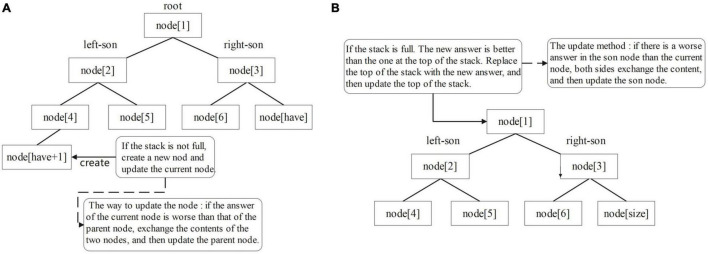
Update process diagram of the min-heap: **(A)** from bottom to top and **(B)** from top to bottom.

Overlapping regions information maintenance method: create a min-heap for each read to maintain the top-k suffix set, the data in the heap store the position of the corresponding suffix in the SA array, and Len stores the matching length between the suffix and the current reads. Node(1) corresponds to the top of the heap. The larger the size of the heap, the more information is obtained and the longer the corresponding program takes to run. To obtain the required information, the top element of the stack is compared with the data in the min-heap, in addition to maintaining the optimal value of the ans array, when traversing the suffix array and encountering a suffix that can obtain the answer [i-fi(da(i)) ≤ k]. If the amount of data in the min-heap is less than k, put the top element of the stack directly into the min-heap and update the heap from the bottom up (Figure 6A). This is because the capacity of the heap is K. There is still space in the heap to store the candidate answers, so the candidate answers can be put directly into the heap and the heap can be updated. Otherwise, compare the top element of the stack with the top element of the heap. If the top element of the heap is better, do not update the elements in the heap; otherwise, use the top element of the stack to replace the top element of the heap and update the heap from top to bottom (Figure 6B). This is because there is no space in the heap to store extra candidate answers, so we must choose between the K answers in the heap and the current candidate answers and delete the worst quality answer. Using this method, software users can obtain more overlapping regions information, which makes the edge weight graph based on this overlap information more high-quality information to facilitate subsequent software processing.

### Output the Final Overlapping Regions Result

The SLDMS software builds an edge-weight graph with read as the point and overlapping regions information as the edge based on the overlap information after obtaining the overlapping regions information, which contains the overlap position information of the two reads in addition to the length of the overlap for use in obtaining the initial contig. Each path in the graph is stitched into a longer read according to the overlapping regions information, which is the initial contig and the SLDMS software stores all the information in this edge weight graph into a file for the next step of obtaining the contig.

When processing reads without mismatch, the SLDMS only counts the overlapping regions information when a complete read is encountered, a complete read is only encountered once, and the result is not updated again in the subsequent maintenance process, so the output to the file is in the order of the encountered reads, which is a way to update the data processing and the result output synchronously. When processing reads with mismatch, the SLDMS collects data for a read several times in the process of maintaining the monotonic stack, so the stored result information may be updated by subsequent maintenance and the program design idea of separating data processing and result output is adopted. To ensure the accuracy of the results, the maintained results are output in the order of the input data after the program has processed all the data.

Although the output of the two strategies is different, logically they both fill in an array of final results and the i-th element of this array stores the answer of the i-th read. The two strategies differ only in the order of filling in the array, one is filling in the array in order and the other is filling in the array in disorder, but the final goal is to fill in the array completely.

### Accuracy Analysis

To prove the universality of the SLDMS software, we write a program to generate the simulated gene sequence randomly, traverse the generated gene sequence many times, and randomly take a substring for simulated gene sequencing. In the first version, the substring is completely correct and in the second version, random errors are generated at both the ends of the substring and used for the SLDMS software input. At the same time, we record the start and end positions of each read and store them in the check file for the final accuracy test.

We consider that the result of each read is correct when it refers to its adjacent read in the gene sequence. After using the SLDMS software to run these input data, the output file and check file are combined to prove the accuracy. According to the results in the output file, we determined whether there was a common part in the interval of the two reads in the check file. If there is a common part, it means that the two reads should be assembled together. We think this is the correct result. We write a program to do this work. First, the program reads the output file and the check file; find each pair of reads and the corresponding overlapping regions information in the output file (if the overlap length is less than 100, it is regarded as invalid data and discarded directly) and then find the corresponding interval in the check file and check the interval. If there is a common part in the two intervals, we can find the corresponding interval in the check file, which is considered the correct result. After calculation, the accuracy of the two versions of the SLDMS is above 99.99%.

The SLDMS software itself and the program code used in the above accuracy proof process are stored on the GitHub website at https://github.com/Dongliang-You/sldms.

## Results

The SLDMS and Flye and Canu software were tested on 6 PacBio-HiFi datasets of different sizes and sequenced species and 32 simulated datasets (16 ultrahigh-accuracy datasets and 16 datasets with errors at both ends of the read) on a desktop computer with an Intel Core (TM) i7-9700 CPU (3.00 GHz 8-core processor), 32 GB RAM, and 477 GB hard disk. Due to the limited hardware conditions of the test environment, the oversized dataset was cut where the descriptions of the PacBio-HiFi dataset are shown in Table 1, which are the datasets downloaded from the official National Center for Biotechnology Information (NCBI) website. The Sequence Read Runs (SRR) in the description represents the data record of the dataset on the website and the specific data information can be viewed at the official website of the NCBI according to the data record information, which is located at https://www.ncbi.nlm.nih.gov/. The data information of the simulated dataset is shown in Supplementary Tables 1, 2.

**TABLE 1:** The PacBio-HiFi dataset used in the experiment and its description.

Dataset	Size(MB)	Description
*Z. mays*	579	WGS of *Zea mays* “B73” using PacBio HiFi Sequencing(SRR11606869)
*E_coli*_K12	1,914	WGS of *E. coli* K12 with PacBio HiFi DNA sheared on Megaruptor to 20 kb(SRR10971019)
*F. × ananassa*_part1	1,131	WGS of *Fragaria × ananassa* Royal Royce using PacBio HiFi Sequencing (SRR11606867)
*F. × ananassa*_part2	2,190	WGS of *Fragaria × ananassa* “Royal Royce” using PacBio HiFi Sequencing(SRR11606867)
*M. musculus*_part1	1,567	WGS of *Mus musculus* “C57/BL6J” using PacBio HiFi Sequencing(SRR11606870)
*M. musculus*_part2	2,760	WGS of *Mus musculus* “C57/BL6J” using PacBio HiFi Sequencing(SRR11606870)

The timing in the experiment starts when the sequencing data are read and ends when the contig is ready to be acquired. This means that it is necessary to be able to build the edge-weight graph from the overlapping regions information to obtain the contig.

For the SLDMS versions that do not allow for mismatching, six PacBio-HiFi datasets with 16 high-accuracy simulated datasets were chosen for testing and comparison with both the Canu and Flye software. When running the Flye software, the genomeSize parameter is the best estimate according to the uasge.md documentation of the Flye software, which is approximately 1% of the file size. The min-overlap parameter is set to 1,000 and the rest of the parameters are the default parameters. When running the Canu software, the genomeSize parameter is the best guess value entered according to the usage documentation, which is the same as the genomeSize parameter of the Flye software and the rest of the parameters are default values without any restrictions.

**FIGURE 7:**
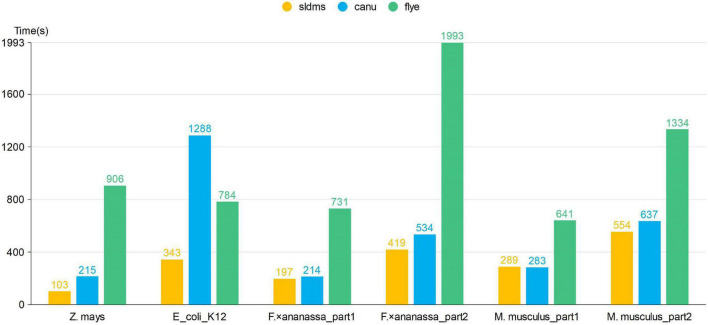
Time required for different software programs to run PacBio-HiFi datasets to find the overlapping regions.

The time required for different software programs to run the PacBio-HiFi datasets to find the overlapping regions is given in Figure 7 and Supplementary Table 3. The SLDMS software ran faster than Flye on all the datasets, faster than Canu on most datasets, and only slightly slower than the Canu software on the *M. musculus*_part1 dataset due to the nature of the algorithm of the Canu software, which makes it potentially efficient at running certain datasets. This result suggests that the SLDMS software runs more efficiently than Canu and Flye for sequence alignment on most PacBio-HiFi datasets.

**FIGURE 8:**
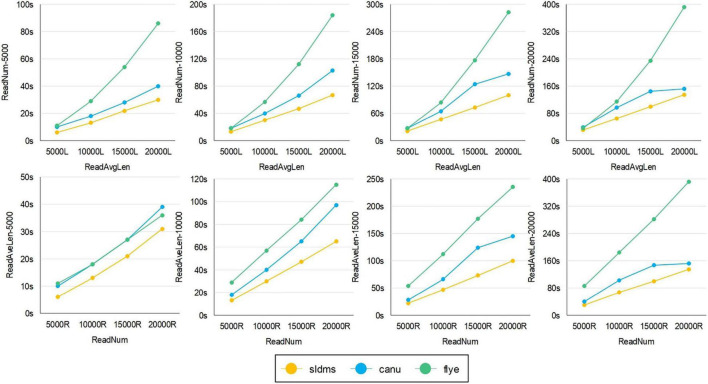
Time required for different software programs to run ultrahigh-accuracy simulation datasets to find overlapping regions.

**FIGURE 9:**
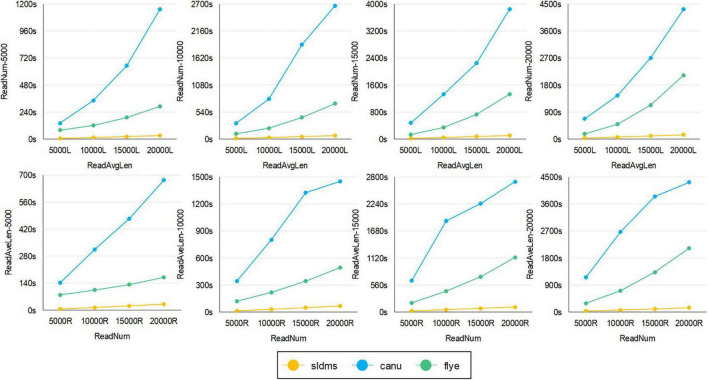
The time required for different software to run simulation datasets with errors to find overlapping regions.

The time required for different software programs to run ultrahigh-accuracy simulation datasets to find overlapping regions is given in Figures 8, 9 and Supplementary Table 4. As shown in the two line graphs in the first row of Figure 8, these are the results of running the ultrahigh accuracy simulation dataset with different average lengths for the same data volume of 5,000, 10,000, 15,000, and 20,000 reads. As shown in the two line graphs in the second row of Figure 8, these are the results of running the ultrahigh-accuracy simulation dataset with different data volumes for the same average data lengths of 5,000, 10,000, 15,000, and 20,000. As seen from the graphs in all the runs, the SLDMS software has a shorter run time than the other two, which suggests that in most cases, it is a good choice to use the SLDMS software to find the overlapping regions information.

For the SLDMS versions that allow for mismatching, there are no real data available that match the conditions for this version to run, so it was only possible to test this version using simulated data. Each read in the simulated data was divided into three parts in order, with the first and third parts being 80% accurate, the second part being 100% accurate, and the second part being at least half the length of the read. The Canu and Flye software selected PacBio-Raw for the data type during the tests and the rest of the parameter settings were the same as the previous version.

The time required for different software programs to run the simulation datasets with errors to find overlapping regions is given in Figure 9, in which row 1 is an experiment with the average length of the data as the variable and row 2 is an experiment with the amount of data as the variable. As seen from the graph, when running these datasets, both when running datasets with different average lengths for the same amount of data and when running datasets with different amounts of data for the same average length, the SLDMS runs much faster than the other two. This shows that using the SLDMS to run this dataset with errors only at both the ends of the data is much better and takes less time than using the other two software tools.

In the experiment, we tested the performance of the different software programs on different datasets. The SLDMS software was stable and efficient in obtaining overlapping regions information for the various test data. The running time of the SLDMS is only related to the size of the input dataset and does not fluctuate depending on differences in the accuracy of the data. This means that the SLDMS is suitable for processing a wide variety of data without the worry that the SLDMS will take a particularly long time to process a particular type of data.

The SLDMS software uses the gsufsort algorithm to calculate the three arrays of SA, LCP, and DA information, which takes a significant amount of time. If new methods are developed to obtain this information more quickly, the SLDMS software will be more efficient.

## Discussion

The main contribution of the proposed method SLDMS for extracting information about the overlapping regions between sequences based on suffix arrays and monotonic stacks is to substantially improve the time efficiency of calculating the overlapping regions. Obtaining overlapping regions information is useful in many bioinformatics applications. As the price of sequencing technology decreases and genome sequencing technology develops, it becomes easier to obtain sequencing data with a wide range of characteristics and higher accuracy. When assembling these sequencing data, it is essential to efficiently extract overlapping regions information between sequences to provide a more favorable environment for subsequent genome assembly work.

The Flye software uses the ABruijn algorithm, which combines the OLC and DBG algorithms to generate its own unique ABG graph, obtains the overlapping regions information of nodes in the graph, and then processes the ABG graph to obtain contigs. In this process, it takes considerable time to process the graph, so we can see from the experimental results that the Flye software takes about twice as long to run as the SLDMS on almost all the datasets.

The Canu software uses the MHAP algorithm to aggregate reads with the same k-mer for error correction and pruning and then obtains the overlapping regions information. When dealing with high-accuracy data without error correction, the SLDMS is around 20% faster than Canu. With respect to error correction, the Canu software is very slow, regardless of the error characteristics of the data, indicating that the Canu software does not take full advantage of the error characteristics of the data. The SLDMS does a very good job in this respect and in some data with most sequencing errors occur at both the ends of the sequencing data, the SLDMS runs at many times the speed of Canu.

The SLDMS obtains the three arrays of SA, LCP, and DA by processing the input data and quickly finds the overlapping regions information in the input data with the help of these three arrays and the monotonic stack. The calculation speed of the overlapping regions information is improved. The experimental results show that compared with the other two kinds of software, the SLDMS have faster speed in the calculation of the overlapping regions and with the help of the SA array containing all the suffixes, it also has the ability of data fault tolerance by cutting the suffixes. This shows that the SLDMS is very efficient as an approach based on suffix arrays and monotonic stacks and that suffix arrays are still the ideal data structure for solving the problem of calculating overlapping regions of gene sequences.

## About the SLDMS

The SLDMS is an open source software tool developed in C and can only be run on Linux systems. The link to the project is (https://github.com/Dongliang-You/sldms). Permission from the author is required before use for non-academic purposes.

## Data Availability Statement

The datasets presented in this study can be found in online repositories. The names of the repository/repositories and accession number(s) can be found below: https://bioinfor.nefu.edu.cn/chenyu/sldms_web/, sldms.

## Author Contributions

YC: conceptualization. DY: software. GW: writing – original draft. TZ: writing – review and editing. All the authors conducted experiments and read and agreed to the published version of the manuscript.

## Conflict of Interest

The authors declare that the research was conducted in the absence of any commercial or financial relationships that could be construed as a potential conflict of interest.

## Publisher’s Note

All claims expressed in this article are solely those of the authors and do not necessarily represent those of their affiliated organizations, or those of the publisher, the editors and the reviewers. Any product that may be evaluated in this article, or claim that may be made by its manufacturer, is not guaranteed or endorsed by the publisher.
