# Supplementary material for: SLDMS: A Tool for Calculating the Overlapping Regions of Sequences
Source: Front Plant Sci. 2022 Jan 3;12:813036. doi: 10.3389/fpls.2021.813036 (PMC8761809; doi:10.3389/fpls.2021.813036)
Supplement: Supplementary file 1 [file Data_Sheet_1.zip › Supplementary_Material_Figure+Table captions.docx]

Supplementary Material

# Supplementary Figures


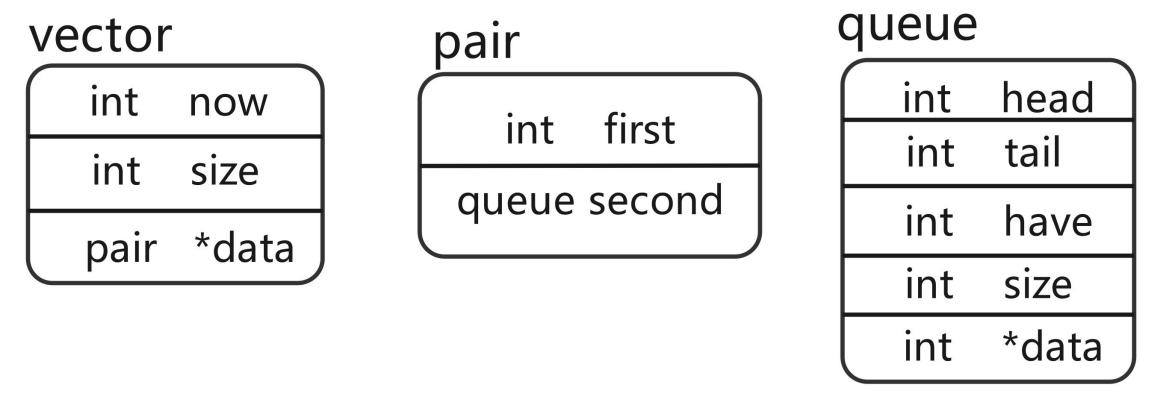


**Supplementary figureS1.** The struct design of the monotone stack and stack elements.

**
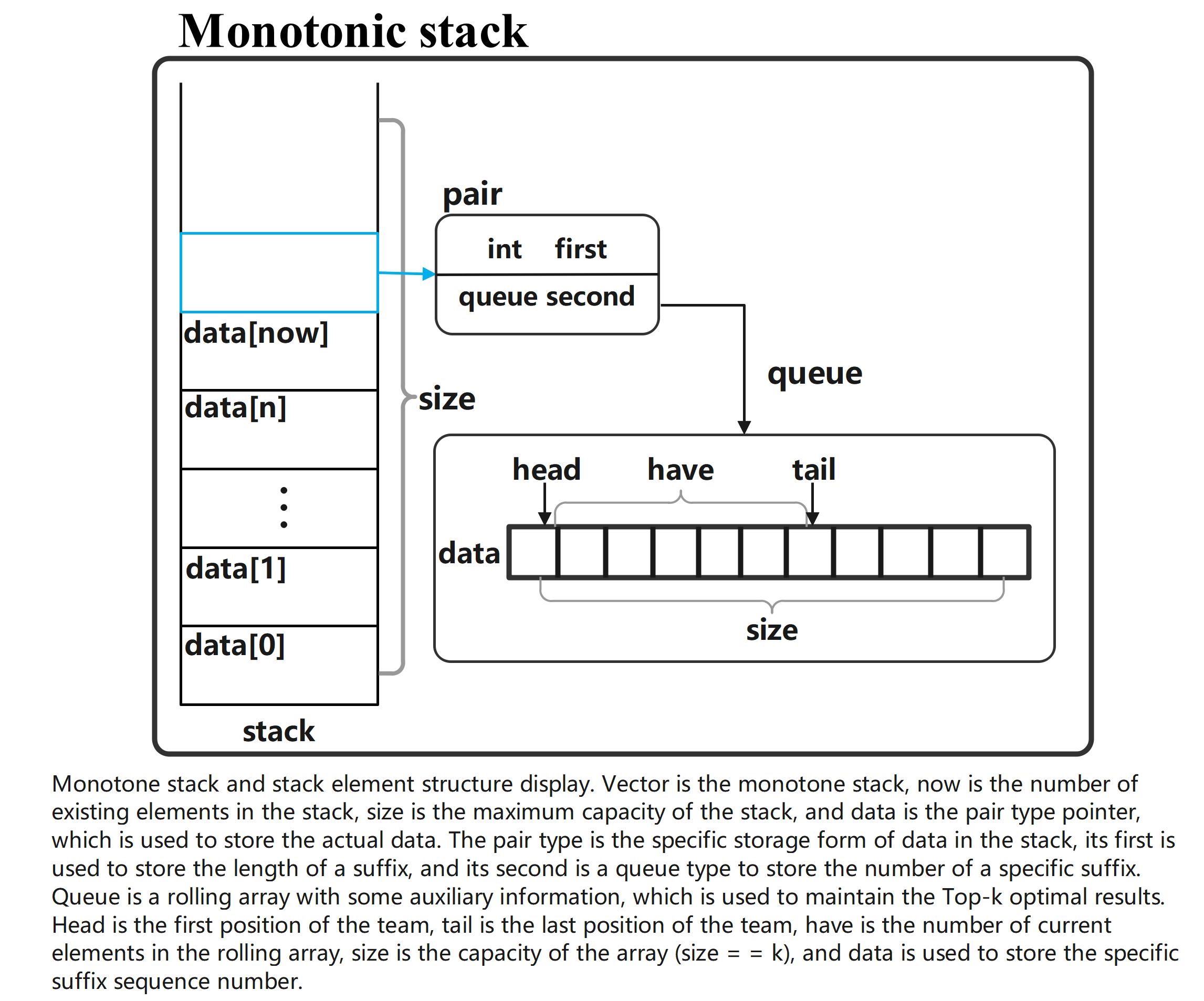
**

**Supplementary figureS2.** Monotonic stack structure for dealing with reads without mismatch.


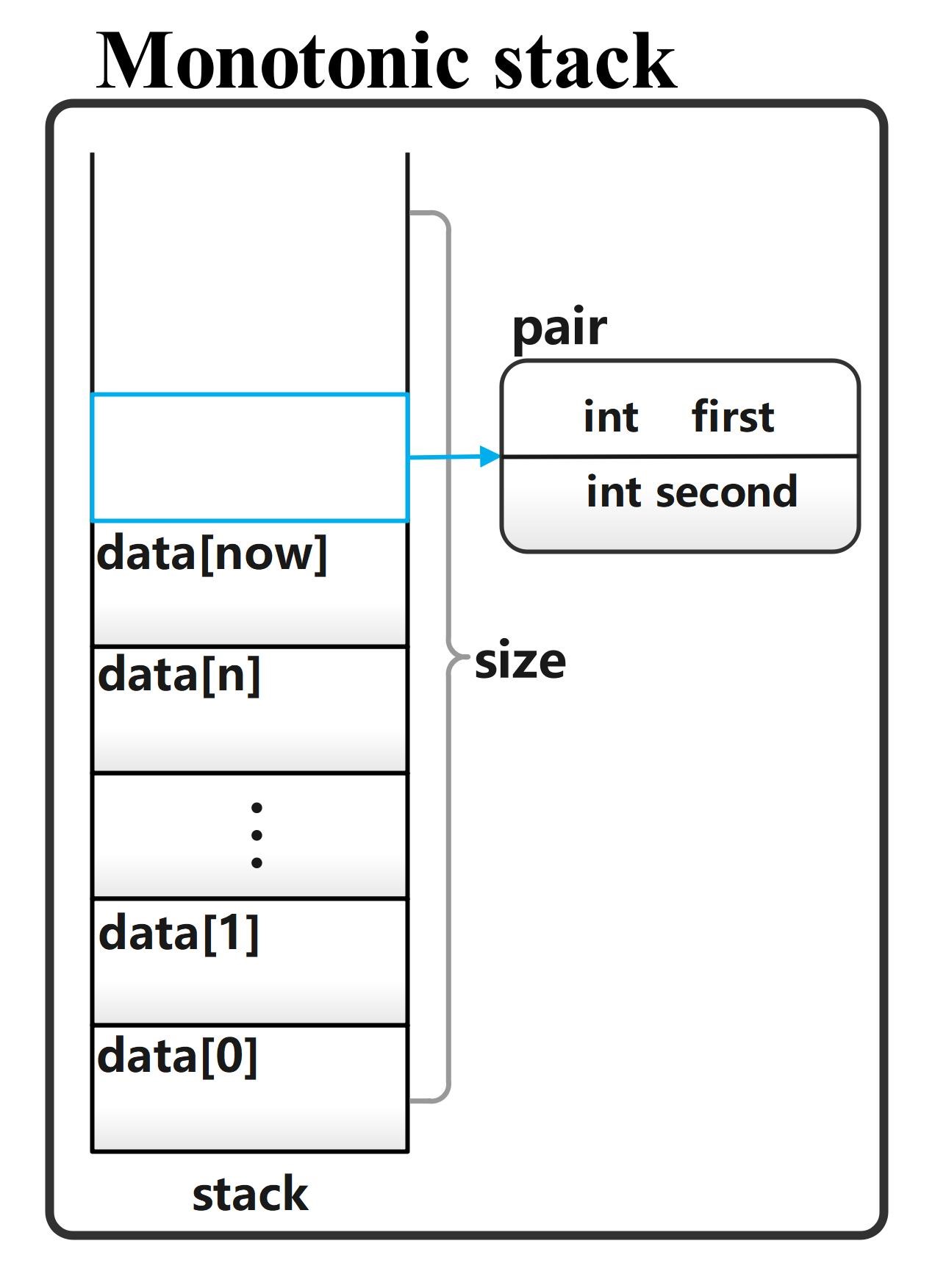


**Supplementary figureS3.** Monotonic stack structure for dealing with reads with mismatch.


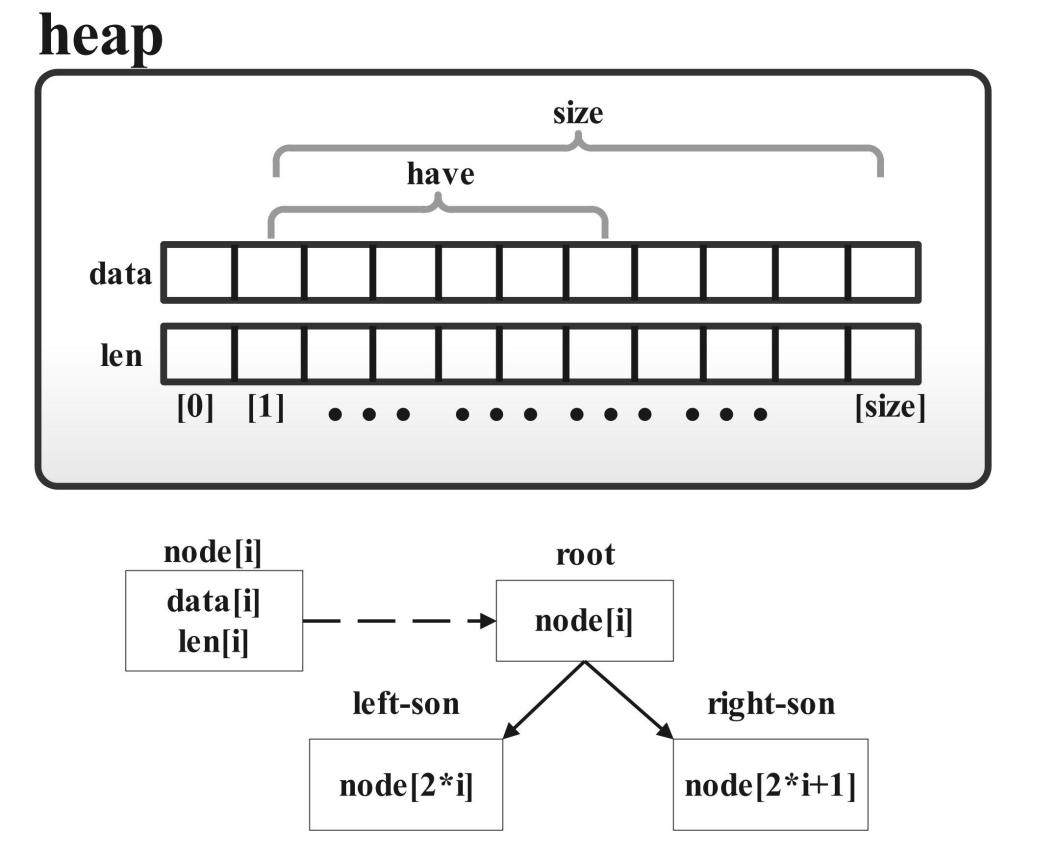


**Supplementary figureS4.** The struct design of the min stack.

# Supplementary Tables

Since the table is too large, only a brief introduction to the table is given here. The specific information of the tables can be found in the corresponding documents.

**Supplementary table T1.** The data information of the correct simulated dataset.

**Supplementary table T2.** The data information of the raw simulated dataset.

**Supplementary table T3.** Time required for different software programs to run pacbio-hifi datasets to find the overlapping regions.

**Supplementary table T4.** Time required for different software programs to run ultrahigh accuracy simulation datasets to find overlapping regions.

**Supplementary table T5.** The time required for different software to run simulation datasets with errors to find overlapping regions.
